# Pyroptosis is a critical inflammatory pathway in the placenta from early onset preeclampsia and in human trophoblasts exposed to hypoxia and endoplasmic reticulum stressors

**DOI:** 10.1038/s41419-019-2162-4

**Published:** 2019-12-05

**Authors:** Shi-Bin Cheng, Akitoshi Nakashima, Warren J. Huber, Sarah Davis, Sayani Banerjee, Zheping Huang, Shigeru Saito, Yoel Sadovsky, Surendra Sharma

**Affiliations:** 1 Departments of Pediatrics, Obstetrics and Gynecology and Pathology, Women and Infants Hospital of Rhode Island, Warren Alpert Medical School of Brown University, Providence, RI USA; 2 Department of Obstetrics and Gynecology, Faculty of Medicine, University of Toyama, Toyama, Japan; 3 Magee-Womens Research Institute, Department of Obstetrics and Gynecology and Reproductive Sciences, University of Pittsburgh, Pittsburgh, PA USA

**Keywords:** Mechanisms of disease, Inflammasome

## Abstract

Systemic manifestation of preeclampsia (PE) is associated with circulating factors, including inflammatory cytokines and damage-associated molecular patterns (DAMPs), or alarmins. However, it is unclear whether the placenta directly contributes to the increased levels of these inflammatory triggers. Here, we demonstrate that pyroptosis, a unique inflammatory cell death pathway, occurs in the placenta predominantly from early onset PE, as evidenced by elevated levels of active caspase-1 and its substrate or cleaved products, gasdermin D (GSDMD), IL-1β, and IL-18. Using cellular models mimicking pathophysiological conditions (e.g., autophagy deficiency, hypoxia, and endoplasmic reticulum (ER) stress), we observed that pyroptosis could be induced in autophagy-deficient human trophoblasts treated with sera from PE patients as well as in primary human trophoblasts exposed to hypoxia. Exposure to hypoxia elicits excessive unfolded protein response (UPR) and ER stress and activation of the NOD-like receptor pyrin-containing 3 (NLRP3) inflammasome in primary human trophoblasts. Thioredoxin-interacting protein (TXNIP), a marker for hyperactivated UPR and a crucial signaling molecule linked to NLRP3 inflammasome activation, is significantly increased in hypoxia-treated trophoblasts. No evidence was observed for necroptosis-associated events. Importantly, these molecular events in hypoxia-treated human trophoblasts are significantly observed in placental tissue from women with early onset PE. Taken together, we propose that placental pyroptosis is a key event that induces the release of factors into maternal circulation that possibly contribute to severe sterile inflammation and early onset PE pathology.

## Introduction

Preeclampsia (PE) is a multifactorial human pregnancy syndrome characterized by de novo onset of hypertension after 20 weeks of gestation with subtypes of early (delivered before 34 weeks) and late onset (delivered after 34 weeks) diagnosis^1–6^. It is a leading cause of maternal and fetal morbidity and mortality, as well as a pivotal risk factor for chronic diseases, including cardiovascular disease, diabetes mellitus, and renal disease later in life^1–12^. Although the etiology of PE is still enigmatic, models that include poor placentation, oxidative stress, and altered local and systemic immune regulation are thought to be a part of the pathophysiology of PE. At the maternal–fetal interface, the unscheduled and excessive placental endoplasmic reticulum (ER) stress arising from hypoxic/ischemic microenvironment leads to release of pregnancy-incompatible factors, such as anti-angiogenic soluble Fms-like tyrosine kinase-1, soluble endoglin, inflammatory cytokines, and alarmins (e.g., cell-free fetal DNA, toxic protein aggregates, and uric acid) into the maternal circulation^5,13–20^. These released factors and alarmins can elicit oxidative stress, systemic inflammation, and endothelial dysfunction as part of the composite PE manifestation profile^17,18^. We have previously shown that protein aggregates present in PE serum can induce PE-like features in a mouse model^19^. We have also demonstrated that protein aggregates are present as part of exosomal cargo in PE placental tissue^20^. However, the tissue origin and the pathways that contribute to circulating alarmins and inflammatory cytokines in PE patients remain poorly understood.

In healthy pregnancy, regulatory mechanisms at the maternal–fetal interface prevent excessive local and systemic inflammation. However, in PE, these regulatory mechanisms are disrupted as a result of local hypoxia/ischemia, innate immune activation, hormonal imbalance, and regulatory T-cell abnormalities. This leads to local cytotoxic microenvironment causing placental inflammation and insufficiency. Although increased noninflammatory apoptotic cell death has been documented in the PE placenta, it is not clear what pathways program placental and systemic inflammation in PE. This is an important issue, particularly in the absence of bacterial and viral infections. Recently, expression of the NOD-like receptor pyrin-containing receptor 3 (NLRP3) inflammasome has been documented in the placenta and in human trophoblasts and monocytes exposed to DAMPs such as high-mobility group box 1 (HMGB1), uric acid, and hyaluronan^21–23^. Interestingly, it has been proposed that extracellular vesicles trigger PE-like phenotype via inflammasome activation in trophoblasts^20,24^. Thus, it is possible that the placenta may release detrimental DAMPs into the circulation. However, a cell death pathway(s) that is associated with inflammasome activation in PE remains not fully understood.

Necroptosis, characterized by activation of mixed lineage kinase domain-like protein (MLKL), and pyroptosis, involving activation of its executor gasdermin D (GSDMD), are pathways that lead to distinct inflammatory cascades causing production and release of alarmins or DAMPs. Necrosis and necroptosis have been described as cell death pathways in trophoblasts in response to high doses of toxic substances^16,21^. Pyroptosis is an inflammatory programmed necrosis that was first described in macrophages infected with *Salmonella*^22^. Unlike other cell death modes, pyroptosis is characterized by NLRP3 inflammasome-promoted and caspase-1-dependent plasma membrane rupture and release of DAMPs and cytokines such as IL-1β and IL-18 into the extracellular milieu, leading to sterile inflammation^22,23,25–31^. Pyroptosis-induced sterile inflammation has been associated with a variety of disorders^22,23,25,26^. We hypothesize that pyroptosis occurs in the trophoblast layer of the PE placenta, which may contribute to release of alarmins for systemic manifestation of the PE syndrome.

This study was designed to determine the pathways related to intrinsic pyroptosis in the placenta from PE pregnancies, including both early and late onset PE cases. To validate the results with the PE placenta, we leveraged ER stress cellular models to further examine signaling pathways that contribute to induction of pyroptosis in human trophoblasts. We show that GSDMD and its signaling pathway proteins are significantly expressed in the early onset PE (e-PE) placenta and in cellular models of PE pathophysiology. We propose that placental pyroptosis is a major sterile inflammatory pathway in e-PE that may lead to production of causative factors such as IL-1β and IL-18, which cause inflammation and potentiate the systemic manifestation of the syndrome.

## Materials and methods

### Human subjects

Diagnosis of PE with severe features was made with strict adherence to previously published guidelines by The Task Force on Hypertension in Pregnancy^32^. PE has mechanistically been classified into e-PE (<34 gestational weeks) and late onset PE (l-PE) (>34 gestational weeks), based on gestational age at diagnosis and/or delivery^33,34^. Demographic information of enrolled patients is shown in Supplementary Table S1. Exclusion criteria included chronic hypertension, gestational or pre-existing diabetes, fetal demise, daily tobacco use, fetal anomalies, and multiple gestations. For each study participant, 7–9 ml of blood was collected in BD Vacutainer SST™ tubes and processed for serum isolation within 30 min. Serum samples were aliquoted in smaller volumes and stored at −80 °C until further use. In cases with pregnancies <34 weeks gestation (either PE patients or gestational age-matched preterm birth controls), all blood samples were collected prior to steroid administration for fetal lung maturity. For placental samples collection, a 1-cm^3^ specimen was removed from the placenta and vigorously washed with chilled phosphate-buffered saline solution. After removing any additional blood from placental tissue, a portion was stored at −80 °C until further use; the remaining was fixed in 10% formalin for immunohistochemical analysis. The study was approved by the Institutional Review Boards at Women and Infants Hospital, Providence, RI and the University of Pittsburgh, Pittsburgh, PA. Informed consent was obtained from all subjects.

### Antibodies and reagents

The following antibodies were used: rabbit anti-TXNIP (Cell Signaling, #14715), rabbit anti- PERK (Cell Signaling, #5683), rabbit anti-IL-18 (Abcam, ab68435), rabbit anti-NLRP3 (Abcam, ab214185), rabbit anti-IL-1β (Santa Cruz, sc-7884), rabbit anti-caspase-1 (Cell Signaling, #2225), mouse anti-Gasdermin D (Abcam, ab57785), mouse anti-β-actin (Cell Signaling), rabbit anti-BiP (Abcam, ab21685), rabbit anti-IRE1a (Abcam, ab48187), rabbit anti-phospho-MLKL (Ser358) (Cell Signaling, D6H3V), rat anti-MLKL (Millipore, MABC604), mouse anti-ASC (B-3) (Santa Cruz, sc-514414), rabbit anti-cleaved gasdermin D (Asp275) (Cell Signaling, #36425), goat anti-rabbit or mouse HRP-conjugated IgG (Cell Signaling), Alexa Fluor 488 donkey anti-rabbit or mouse IgG (Molecular Probes, A-21206), and Alexa Fluor 594 donkey anti-mouse IgG (Molecular Probes, A-21203). The following reagents were used: STF 083030 (IRE1a inhibitor, Santa Cruz, CAS 307543-71-1); resveratrol (TXNIP inhibitor, Sigma, R5010-100MG); PERK inhibitor II (Sigma, 5046510001); NLRP3 inhibitor (Sigma, 5381200001).

### Generation of autophagy-deficient trophoblasts

An autophagy-deficient cell line (named Atg4BC74A) was generated using HchEpC1b cell line, an HPV E6 and hTERT-transfected immortalized extravillous trophoblasts as previously described^35^. Briefly, HchEpC1b cells were stably transfected with pMRX-IRES-puro-mStrawberry-Atg4B^C74A^, an Atg4B^C74A^ mutant expression vector that inhibits MAP1LC3B-II formation^35^. For control, HchEpC1b cells were stably transfected with pMRX-IRES-puro-mStrawberry, a control vector only encoding monomeric red fluorescent protein^35^. After transfection, the cells were grown in the RPMI1640 medium (GIBCO, 11875, MA, USA) supplemented with 10% FBS and selected by addition of 0.3 μg/ml puromycin (Sigma, P8833) in the medium.

### Cell culture and treatment with ER stressors

Autophagy-deficient trophoblast cells and human primary trophoblasts (purchased from ScienCell Research Laboratories, Carlsbad, CA) were cultured in the RPMI1640 medium (GIBCO, 11875, MA, USA) supplemented with 10% FBS, 100 U/ml penicillin, and 100 µg/ml streptomycin (GIBCO, 15140) at 37 °C in a 5% CO_2_ atmosphere. Cells were seeded at 400,000 cells/well onto glass coverslips in 6- or 12-well plates and washed three times in serum-free media, incubated overnight or for 24 h with 10% sera from PE or normal pregnancy, brefeldin A (2.5 μg/ml, Cell signaling), or chloroquine (50 μM, Sigma-Aldrich), and then fixed with 4% paraformaldehyde in PBS buffer for 10–15 min for immunofluorescent staining.

### Hypoxia treatment

Primary human trophoblasts (PHT cells) were isolated from placental tissue from three different women with singleton normal pregnancies at 37–40 gestational weeks or purchased (ScienCell). PHT cells were dispersed using the trypsin–deoxyribonuclease–dispase/Percoll method as described^36,37^ and then seeded at a density of 300,000 cells/cm^2^ and cultured for 4 h in media (DMEM with “high glucose” of 4.5 gm/L D-glucose) with standard culture atmosphere that supports cell differentiation into syncytiotrophoblast^38^. After 4 h, PHT cells were exposed for 72 h to normoxia or a low oxygen tension (1% O2) using a hermetically enclosed incubator (Thermo Electron, Marietta, OH, USA) with continuous digital recording of atmospheric oxygen using a sensor connected to a data acquisition module (Scope; Data Translation, Marlboro, MA, USA). The media was pre-equilibrated to the gas mixture before addition to the culture plate, and refreshed every 24 h. On day 2, PHT cells were treated with vehicle, STF 083030 (IRE1α inhibitor), resveratrol (TXNIP inhibitor), PERK inhibitor II, or NLRP3 inhibitor. The cells were then harvested and lysed on day 3 in RIPA buffer containing 25 mM Tris-HCl, 150 mM NaCl, 1% sodium deoxycholate, 1% NP40, 0.1% SDS, protease inhibitor cocktail (Roche, 0469316001), and 1% phosphatase inhibitor (Sigma-Aldrich, P5726).

### Preparation of placenta homogenates

Placental tissues were washed five times in ice-cold PBS and then homogenized using a homogenizer (USA) in RIPA buffer. Homogenates were centrifuged at 12,000 *g* for 25 min at 4 °C, and supernatant was collected, aliquoted and stored at −80°C until use.

### Immunoblotting

Protein concentration was measured using the BCA assay. Equal amounts of protein extracts were resolved by 10% SDS-PAGE according to standard procedures. After blocking in 5% nonfat milk dissolved in PBS buffer (pH 7.4) containing 0.1% Tween 20 (PBST) for 1 h, the transferred membrane was incubated overnight in primary antibody solution diluted in 5% nonfat milk or 3% BSA in PBST at 4 °C. The membrane was washed three times, incubated for 1 h at room temperature with HRP-conjugated donkey anti-rabbit IgG (Cell signaling), treated with chemiluminescence substrate (SuperSignal, Pierce), and exposed on film (Kodak). Density of blots was measured using ImageJ (NIH).

### Immunofluorescence

For placental tissues, paraffin-embedded sections from gestational age-matched pregnant and preeclamptic women were de-paraffinized, subjected to heating antigen retrieval procedure in sodium citrate buffer (10 mM sodium citrate, 0.05% Tween 20, pH 6.0) and then incubated with 0.1% Sudan Black for 20 min at room temperature to quench autofluorescence. Sections were then incubated overnight at 4 °C with the primary antibodies, diluted in PBS buffer containing 3% BSA, 3% normal donkey serum, and 0.1% Triton X-100. After extensive washing with PBS, the sections were incubated for 1 h with the secondary antibodies. For cell culture, fixed cells were permeabilized in blocking buffer containing 3% BSA, 3% normal donkey serum, and 0.1% Triton X-100 in PBS. Cells were then incubated overnight in primary antibodies diluted in blocking buffer. After several washes, the cells were incubated for 1 h at room temperature in secondary antibodies, washed in PBS, and then mounted in anti-quench mounting medium with DAPI (Vector Laboratories, Inc., Burlingame, CA). Negative controls were performed by replacing the primary antibody with purified rabbit IgG or mouse IgG. Immunofluorescent images were visualized with a fluorescent microscope, Nikon Eclipse TE2000 (Nikon, Tokyo, Japan), and analyzed using MetaVue Imaging software (Molecular Devices, CA, USA). The pixel intensity of immunoreactive signal was measured using ImageJ (NIH). Figures were processed with brightness/contrast adjustment using Photoshop CS2 (Adobe).

### Lactate dehydrogenase (LDH) assay

Primary human villous trophoblasts (ScienCell) were cultured in the trophoblast medium (TM, ScienCell) and exposed to hypoxia or normoxia as described above. The supernatant was collected at day 3 and assessed for LDH activity using LDH Assay Kit (Colorimetric) according to the manufacturer’s instruction (ab102526, Abcam). The optical density values for LDH, normalized to total cell numbers, were compared between hypoxia- and normoxia-treated cells.

### Statistical analysis

The results were presented as the mean ± SD, and comparisons between experimental groups were statistically analyzed using a Student’s *t* test or an ANOVA followed by a post hoc test if *p*-value is significant (SigmaPlot, Systat Software, Inc). Differences between the groups were considered significant when the *p*-value was <0.05.

## Results

### Evidence for the presence of pyroptosis but not necroptosis in the placenta from women with e-PE

**Fig. 1:**
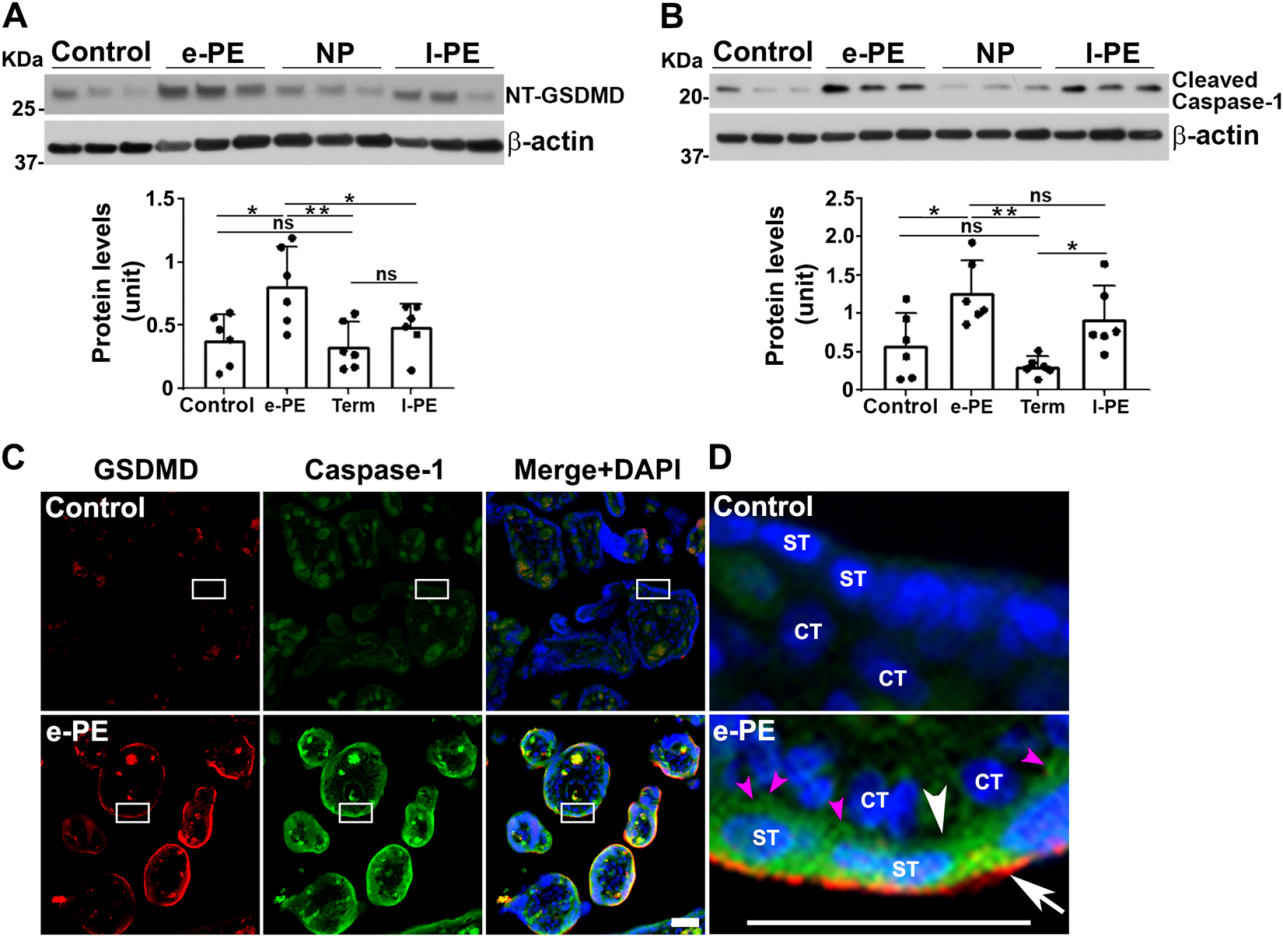
GSDMD and caspase-1 are upregulated in the placenta from early onset PE (e-PE) deliveries. Placental tissues from e-PE, preterm control, l-PE, and NP were homogenized and subjected to western blotting analysis. Fixed placental tissue sections were used for immunohistochemical analysis. Representative experiments are shown. β-actin was used as a protein loading control. **a** Immunoblotting and quantitative analyses show higher levels of cleaved GSDMD protein in the placenta from women with e-PE versus PT (*n* = 6, *p < 0.05, **p < 0.01). No significant alteration of GSDMD were observed in preterm control vs. NP (*n* = 6, *p* = 0.98). **b** e-PE and l-PE placentas exhibit higher levels of cleaved caspase-1 than their corresponding controls (*n* = 6, *p < 0.05, **p < 0.01). No significant alteration of cleaved caspase-1 was observed in PT vs. NP (*n* = 6, *p* = 0.59). **c** Representative immunofluorescent images show higher levels of both GSDMD (red) and caspase-1 immunoreactivity (green) in the trophoblast layer of placental villi from e-PE vs. control placental tissue samples (*n* = 6). **d** Magnified images from boxed areas in **c** reveal that robust GSDMD immunoreactivity is predominantly localized to apical surface of syncytiotrophoblasts, and caspase-1 signal is mainly distributed in the cytoplasm of syncytiotrophoblasts (ST) and to lesser extent in cytotrophoblasts (CT) of the placental villi from e-PE. Nuclei were stained with DAPI (blue). Arrow indicates apical surface; white arrowhead indicates basal line of the cells, and pink arrowheads show peck-like caspase-1 staining. These results are representative of at least three independent experiments. Scale bar: 50 μm. Data are presented as mean ± SD and analyzed by a one-way ANOVA.

GSDMD, particularly active N-terminal GSDMD (NT-GSDMD), has been identified as a master executor of pyroptosis^39–44^. First, we determined the abundance of this protein in the placenta from PE vs. control using western blotting. Protein extracts from placental samples from e-PE vs. gestational age-matched preterm deliveries (<34 gestational weeks) and late onset PE (l-PE) vs. normal term pregnancy (NP) (>34 gestational weeks) deliveries (Supplementary Table 1) were subjected to western blotting. As shown in Fig. 1a using three different placentas of each, band intensity and its densitometric analyses revealed a significant increase in the expression of NT-GSDMD (30 kDa) in e-PE placentas after normalization of NT-GSDMD levels to β-actin (Fig. 1a, bottom panel; *p* < 0.05). In contrast, no significant increase or difference was observed for NT-GSDMD between preterm birth and NP samples. A small increase was noticed in NT-GSDMD in l-PE samples, but this increase was not statistically significant when compared with e-PE placental samples from multiple deliveries (Fig. 1a; Supplementary Fig. 1a, b). To determine whether necroptosis occurs in PE placenta, total MLKL and phosphorylated MLKL (serine358) were examined in placental tissues. The results showed that total MLKL (54 kDa) was present at the same level in e-PE vs. control samples (Supplementary Fig. 2a). However, phosphorylated MLKL (pMLKL) was not easily detectable in any of the placental samples tested, but was detected in known positive control cells treated with caspase inhibitors (Supplementary Fig. 2b). Similar results were also observed in l-PE and NP placentas (data not shown). Since GSDMD is activated by caspase-1 cleavage, we then assessed the expression of caspase-1 in placental samples from preterm birth vs. e-PE and NP vs. l-PE. Similar to GSDMD, caspase-1 is significantly increased in the placenta from e-PE as compared with controls as shown by western blot analyses and densitometry (Fig. 1b; Supplementary Fig. 1c, *p* < 0.05). In contrast, no difference in caspase-1 expression was observed in the placentas from preterm birth women vs. NP controls. However, increased abundance of caspase-1 was also observed in the placenta from l-PE relative to NP (Fig. 1b).

As NT-GSDMD-assembled pore structures on the plasma membrane are characteristic of the pyroptosis pathway, we performed immunofluorescent staining to visualize the localization of GSDMD and caspase-1 in placental tissue sections from e-PE and preterm birth deliveries. As shown in Fig. 1c, robust immunoreactivity of GSDMD appeared to be predominantly localized to the apical surface of syncytiotrophoblasts of the villous trophoblast layer as well as a few other cells in the stroma but not cytotrophoblast cells, whereas speck-like caspase-1 immunoreactivity was observed in the trophoblast layer with marked staining in syncytiotrophoblasts. In contrast, placental tissue from preterm deliveries exhibited only low levels of GSDMD and caspase-1 signal throughout the villous structure. These data confirm the results presented in Fig. 1a, b and provide evidence for the presence of key pyroptosis-associated proteins in placental tissue from e-PE deliveries.

### Increased GSDMD expression and caspase-1 activation can be induced in autophagy-deficient trophoblasts exposed to sera from e-PE women and in primary trophoblasts treated with ER stress inducers

**Fig. 2:**
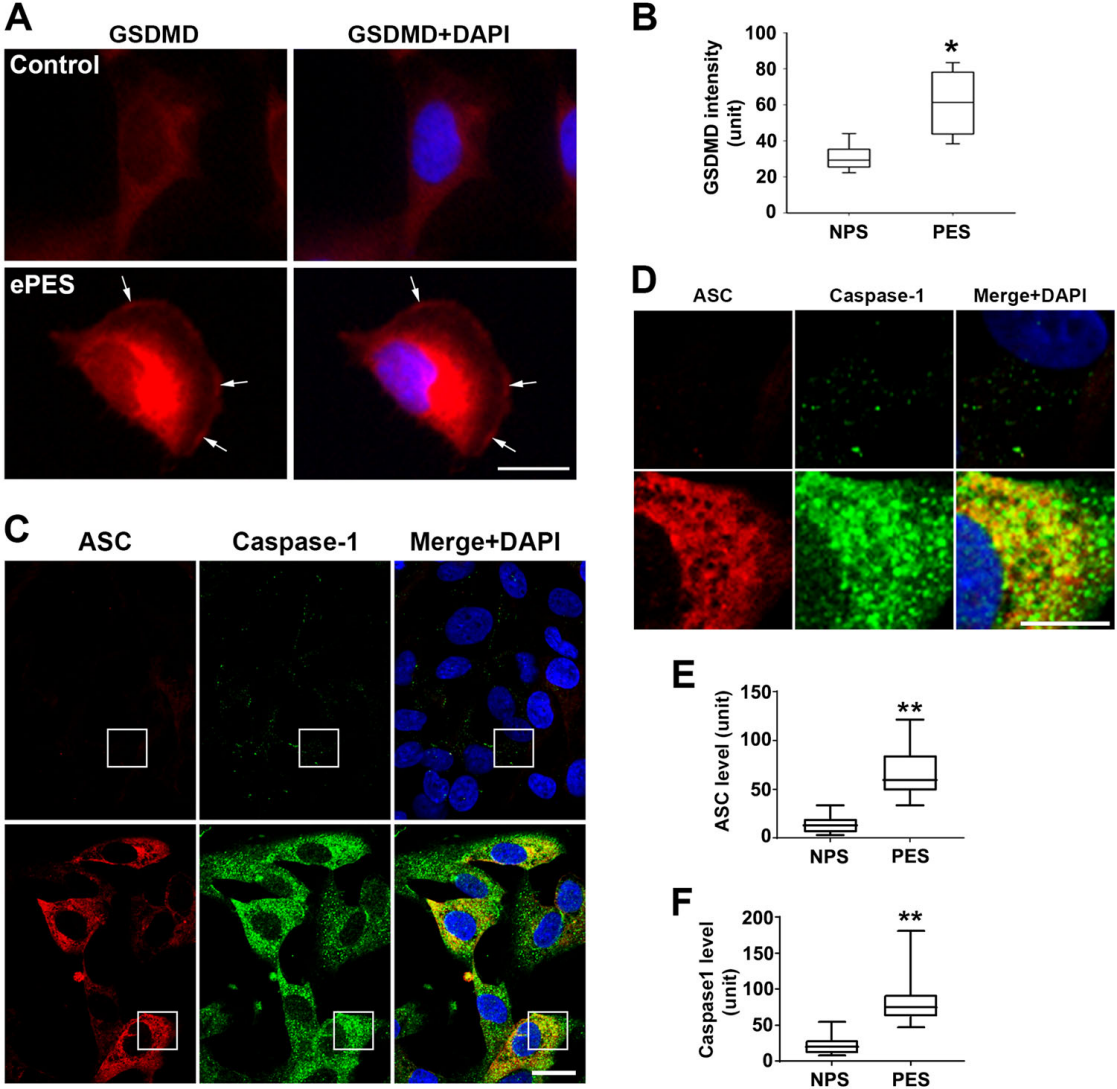
Serum from e-PE, not gestational age-matched pregnancy, increases the expression of GSDMD, ASC and caspase-1 in autophagy-deficient trophoblast cells. Autophagy-deficient cells were treated with sera from e-PE (ePES) or gestational age-matched preterm birth control (control), fixed at 24 h and immunostained for GSDMD, ASC, or caspase-1. Nuclei were stained with DAPI. **a** Representative immunofluorescent images for GSDMD in ePES-treated and control cells. Scale bar: 20 μm. **b** Quantitative analysis shows that GSDMD expression is significantly higher in ePES-treated cells (*n* = 45) vs. control cells (*n* = 60) (*p* < 0.05). Arrows indicate localization of GSDMD to cell surface. **c** Representative confocal microscopy images demonstrate the expression and co-localization of ASC (red) and caspase-1 (green) immunofluorescent signals in ePES-treated and control cells. Scale bar: 20 μm. **d** Magnified images (×4) of boxed areas in **c** show speck-like ASC and caspase-1 staining and co-localization of two proteins (in yellow). Scale bar: 10 μm. **e**, **f**, Quantitative analysis of pixel intensity of ASC and caspase-1 in **c**. Difference in ASC and caspase-1 expression is statistically significant between ePES-treated and control cells (*n* = 55). Images are representatives from three independent experiments. Data are presented as mean ± SD and analyzed by a Student’s *t* test. *p < 0.05. **p < 0.001.

Prior results suggest that autophagy is a crucial regulator of inflammation, and impaired autophagy is associated with the pathogenesis of PE^45,46^. Sera from e-PE (ePES) patients contain protein aggregates that can cause the onset of PE-like features in pregnant mice^47^. Importantly, sera from severe PE can mimic ER stress inducers in that they disrupt endovascular cross talk between human endothelial cells and first trimester trophoblasts and impair autophagy^14,47^. To determine whether ePES induces GSDMD membrane translocation and caspase-1 activation in autophagy-deficient trophoblasts, these cells were exposed to ePES or gestational age-matched pregnancy serum for 24 h and analyzed for cellular distribution of GSDMD and co-localization of caspase-1 with apoptosis-associated speck-like protein containing a caspase-recruitment domain (ASC), a partner for active caspase-1 in the inflammasome complex by immunofluorescence. As shown in Fig. 2a, b, ePES, but not control sera, significantly increased the abundance of GSDMD (*p* < 0.05). Notably, ePES-induced cell surface localization of GSDMD (Fig. 2a) mimics the appearance of GSDMD signals observed in PE placental tissue (Fig. 1c, d). In addition, confocal imaging revealed that speck-like ASC and caspase-1 immunostainings were significantly upregulated in response to ePES but not control (Fig. 2c–f), and that ASC signal co-localized with caspase-1 immunofluorescence, indicative of caspase-1 activation (Fig. 2d).

To directly confirm the effect of ER stress on GSDMD expression in human primary trophoblast cells, we exposed these cells to ER stressors, brefeldin A and chloroquine. The results indicate that these ER stressors significantly augmented the abundance of GSDMD (Supplementary Fig. 3). Taken together, these results suggest that ER stress enhances GSDMD abundance both in the cytoplasm and on the cell surface of trophoblasts, and that ePES mimics ER stress inducer, inducing elevated expression of ASC, caspase-1, and GSDMD in autophagy-deficient trophoblasts.

### Hypoxia (1% O_2_) increases caspase-1, GSDMD, inflammatory cytokines, NLRP3, and cell death in primary trophoblast cells

**Fig. 3:**
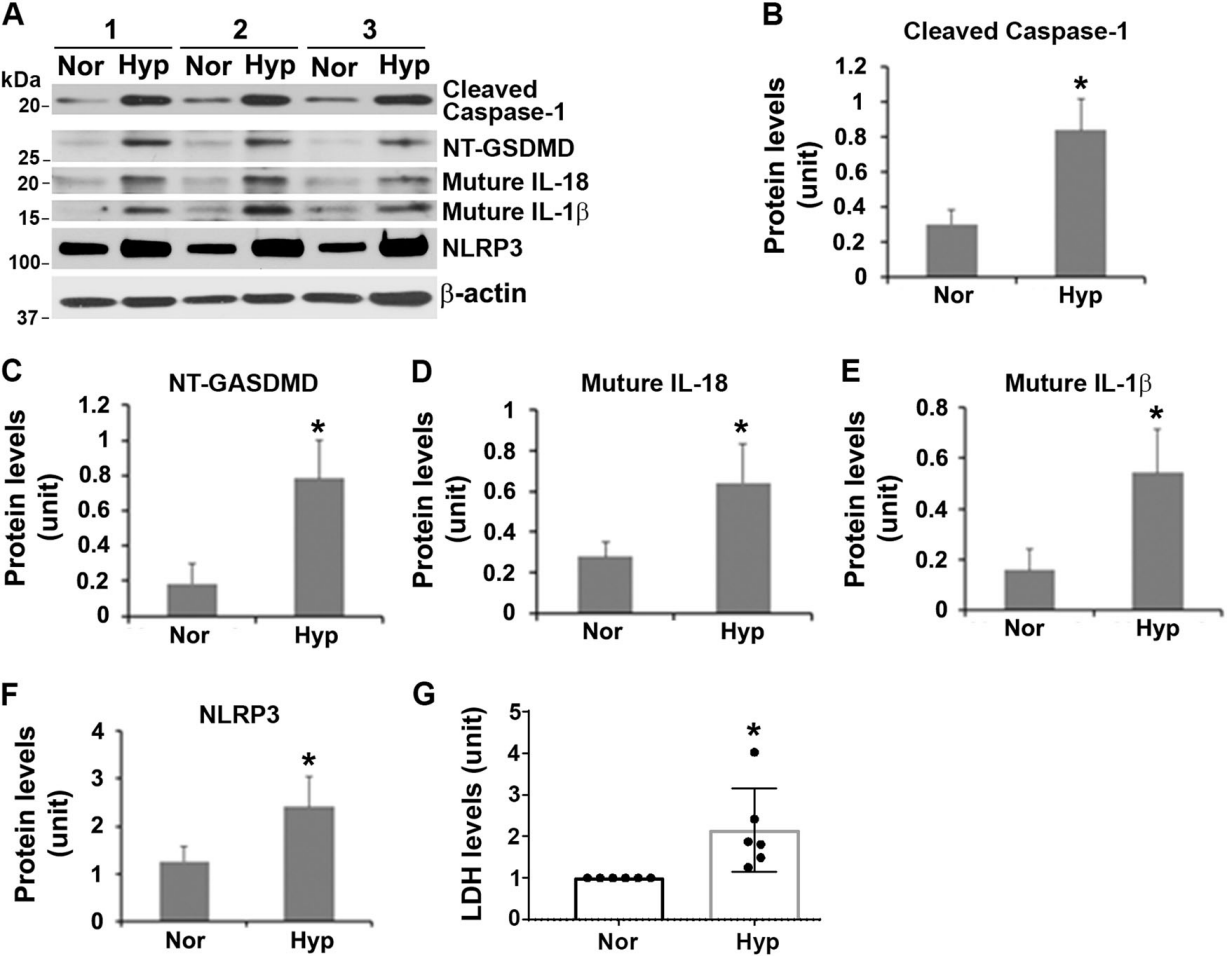
Persistent hypoxia increases signaling molecules in pyroptosis pathway and enhances lactate dehydrogenase (LDH) in human primary trophoblasts. Primary trophoblast cells, isolated from the placentas from three deliveries with normal pregnancy at term, were treated with hypoxia (Hyp) or normoxia (Nor) for 3 days and lysed for immunoblotting. Hypoxia increases the expression of caspase-1, GSDMD, IL-18, IL-1β, and NLRP3. Comparison of the signal intensity reveals that these molecules are significantly elevated in hypoxia-treated primary trophoblasts relative to control cells (*n* = 3, *p* < 0.05). **c** LDH colorimetric assay shows higher level of LDH in supernatant from hypoxia-treated cells vs. control cells (*n* = 6, *p* < 0.05). Data are presented as mean ± SD and analyzed by a Student’s *t* test.

Chronic hypoxic/ischemic microenvironment has been thought to be a major ER stress inducer that contributes to the PE pathology^2,3,13–16^. Next, we investigated whether prolonged exposure to low oxygen tension (1% O_2_, hypoxia) induced the pyroptosis-related signaling cascade in human primary trophoblasts. Freshly isolated trophoblasts from term delivery placental tissue were exposed to 1% oxygen in a regulated hypoxic chamber or atmospheric oxygen tension in an incubator (normoxia) for 3 days. Immunoblotting results presented in Fig. 3a showed that exposure to hypoxia led to a significant increase in the expression of caspase-1 and NT-GSDMD as compared with normoxia controls. Since caspase-1 activates the production of IL-1β and IL-18 by cleaving their precursor counterparts, content of these cytokines was then examined in trophoblasts in response to hypoxia. As expected, both inflammatory cytokines were significantly produced in the hypoxia-treated trophoblasts relative to the normoxia controls (Fig. 3b–e, *P* < 0.05). Since caspase-1 activation is primarily mediated by NLRP3 inflammasome in immune and nonimmune cells^22,23,25–27^, we next tested whether hypoxia can increase NLRP3 abundance in primary trophoblast cells. As shown in Fig. 3a, f, higher levels of NLRP3 were observed in hypoxia-treated cells vs. normoxia-treated cells (*p* < 0.05). These results indicate that hypoxia can increase the content of NLRP3 and NT-GSDMD as well as inflammatory cytokines, IL-1β and IL-18. To further confirm whether hypoxia-treated cells undergo cell death, LDH concentration in culture media was measured and compared between normoxia- and hypoxia-treated primary trophoblasts. Figure 3g demonstrated that hypoxia-exposed cells released more LDH into the culture media than control cells. These findings suggest that pyroptosis can be induced in trophoblasts in response to ER stress mediated by hypoxia.

### Hypoxia hyperactivates UPR and increases TXNIP in human primary trophoblast cells

**Fig. 4:**
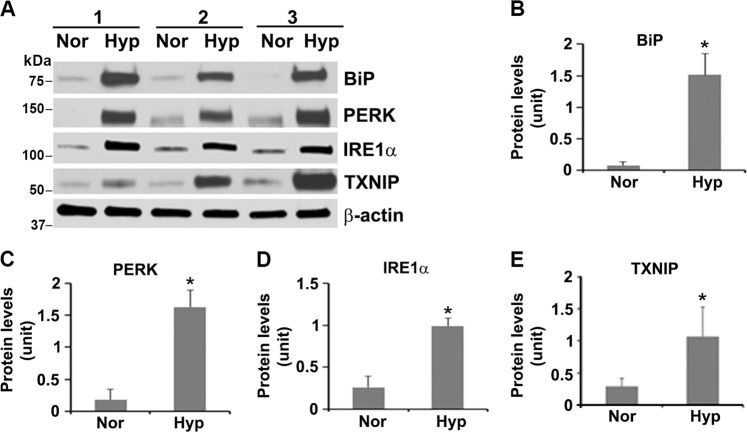
Prolonged hypoxic stimulation induces ER stress, hyperactivates UPR in human primary trophoblast cells. **a** Primary trophoblast cells were exposed to hypoxia (Hyp) or normoxia (Nor), lysed at day 3 and subjected to immunoblotting for BiP, PERK, IRE1α, and TXNIP. **b**–**e** Quantitative analyses of blots indicate significant increase in the expression of BiP, PERK, IRE1α, and TXNIP in hypoxia-treated cells compared with the controls (*n* = 3, *p* < 0.05). Data are presented as mean ± SD and analyzed by a Student’s *t* test.

Next, based on the data described above, we attempted to further understand the molecular events underlying pyroptosis in primary trophoblasts. Excessive UPR activation has been reported to trigger inflammatory events, which, in turn, exaggerate ER stress^48–50^. To evaluate the effect of hypoxia on the UPR activity, we examined the expression of several components of the UPR pathway, including binding immunoglobulin protein (BiP, an ER stress marker), inositol-requiring kinase-1 α (IRE1α) and protein kinase R (PKR)-like endoplasmic reticulum kinase (PERK). Immunoblotting analyses demonstrated that hypoxic stimulation for 72 h induced significant increase in the protein content of BiP, PERK, and IRE1α compared with normoxia controls (Fig. 4, *p* < 0.05).

**Fig. 5:**
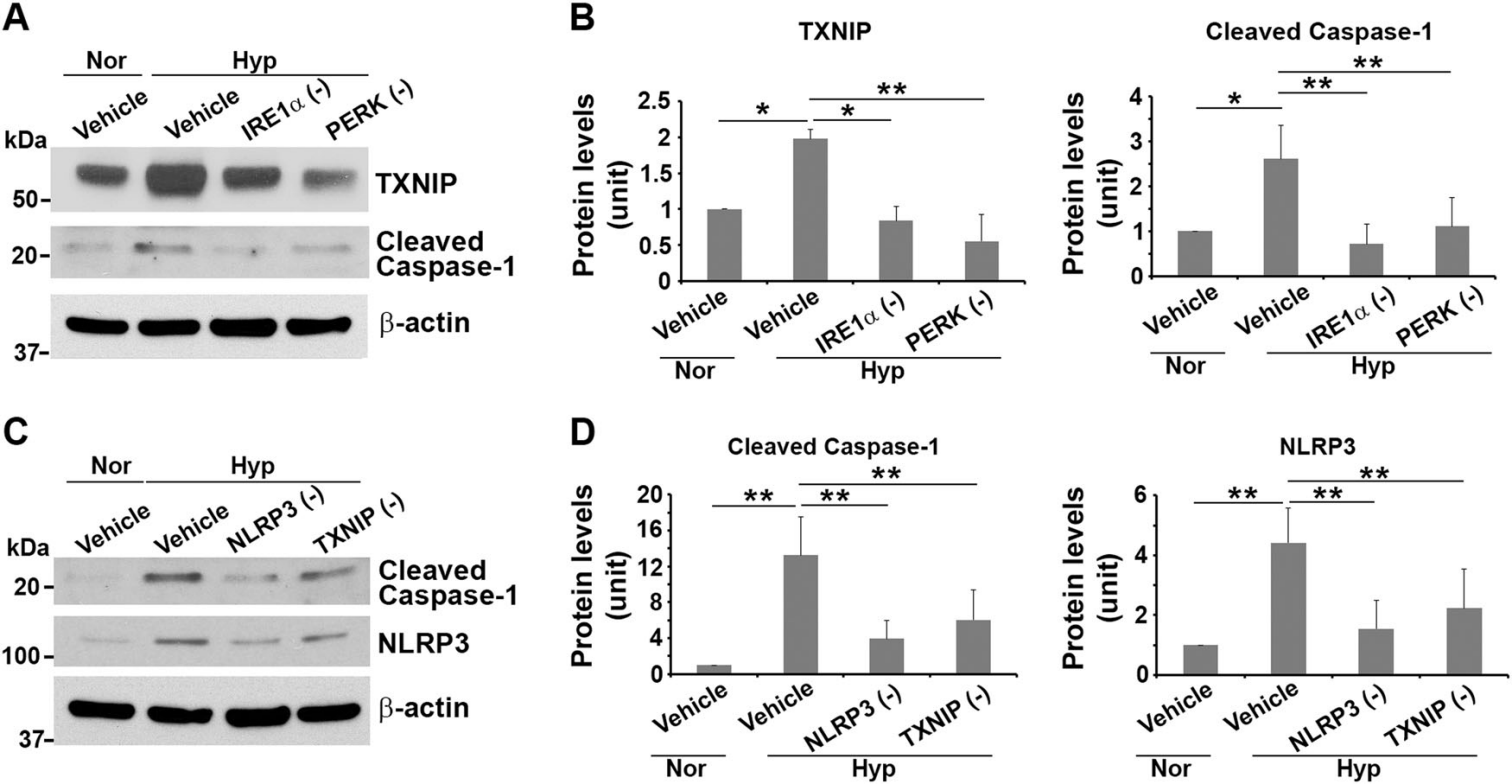
Effect of inhibition of IRE1α, PERK, NLRP3, or TXNIP on the TXNIP-NLRP3-caspase-1 pathway. Primary trophoblasts were exposed to normoxia (Nor) or hypoxia (Hyp), treated with various inhibitors of IRE1α (10 μM), PERK (10 μM), NLRP3 (50 μM), or TXNIP (50 μM) on day 2, and lysed on day 3. Immunoblotting revealed that IRE1α or PERK inhibitor significantly attenuated hypoxia-induced increase in TXNIP and caspase-1 expression (**a**, **b**), and that TXNIP inhibitor remarkably reduced hypoxia-induced abundance of NLRP3 and caspase-1, and NLRP3 inhibitor reduced caspase-1 presence (**c**, **d**). Data are presented as mean ± SD and analyzed by a one-way ANOVA (*n* = 3, *p < 0.05; **p < 0.01).

Recent reports have shown that overactivation of PERK and IRE1α induces elevated expression of TXNIP, which, in turn, activates the NLRP3 inflammasome^50–52^. Thus, TXNIP functions as a regulatory liaison between the UPR machinery and NLRP3 inflammasome activation, thereby serving as a marker for excessive ER stress and UPR activation^50–52^. Based on these observations, we assessed the expression of TXNIP in primary trophoblast cells under hypoxic conditions. As shown in Fig. 4a–e, exposure to hypoxia for 72 h elicited a significant increase in TXNIP expression compared with normoxic controls (*p* < 0.05). Next, we tested whether inhibition of IRE1α and PERK activities were able to reduce TXNIP and caspase-1 abundance in trophoblasts as observed in other cellular systems^50–52^. As expected, treatment of primary human trophoblasts with IRE1α or PERK inhibitor significantly reduced TXNIP and caspase-1 presence in hypoxia-insulted cells compared with control cells (Fig. 5a, b, *p* < 0.05). Moreover, we assessed the existence of the TXNIP-NLRP3-caspase-1 pathway in hypoxia-exposed trophoblasts. To this end, hypoxia-treated primary trophoblasts were incubated with TXNIP or NLRP3 inhibitor. As indicated in Fig. 5c, d, TXNIP inhibitor reduced both NLRP3 and caspase-1 abundance, and NLRP3 inhibitor reduced caspase-1 and NLRP3 presence in hypoxia-exposed cells. These results suggest that chronic hypoxic conditions induce excessive ER stress and UPR, leading to increase in caspase-1 expression through the TXNIP-NLRP3-caspase-1 pathway in primary human trophoblasts.

### Placental tissues from e-PE deliveries show increased expression of UPR proteins, TXNIP and NLRP3

Next, we attempted to extend the observations from hypoxia-exposed human trophoblasts (Figs. 3, 4) to the e-PE placenta. To address this issue, we first examined whether the PE placenta exhibits hyperactivated UPR and augmented presence of TXNIP. If this were the case, increased NLRP3 inflammasome and production of IL-1β and IL-18 should be observed in the placenta from e-PE pregnancies.

**Fig. 6:**
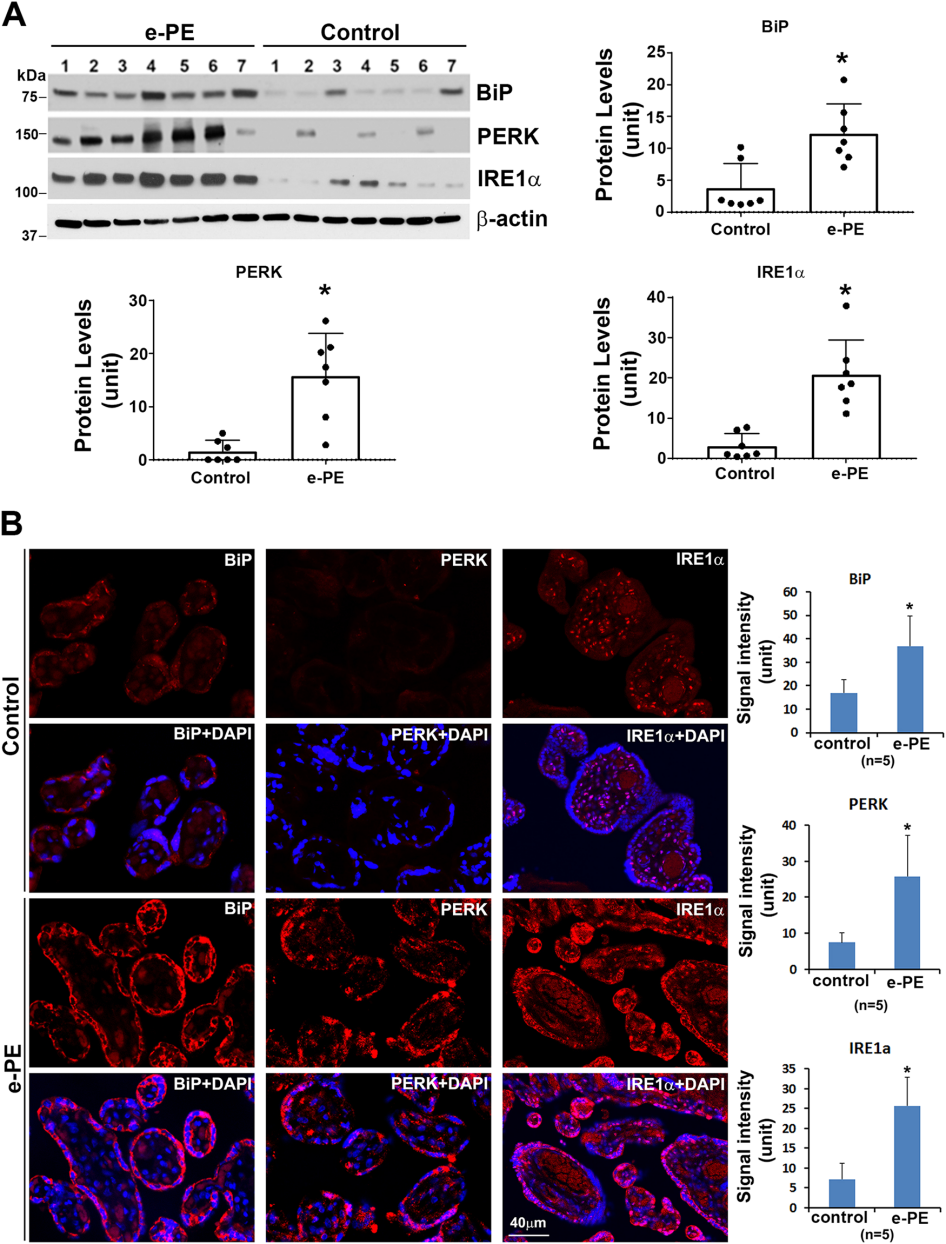
Expression and distribution of BiP, PERK, and IRE1α in e-PE placentas. **a** Placental tissues from e-PE and preterm control were subjected to western blotting and probed for indicated protein molecules. Quantification of the intensity of bands indicates that PE placentas contain higher levels of these molecules compared to the controls (*n* = 7, *p < 0.05). **b** Paraffin-embedded placental section from e-PE and gestational age-matched controls (control) were immunostained for BiP, PERK, and IRE1α. Nuclei were stained with DAPI. Images are representatives from five preeclamptic women and five control women. Quantitative data show that PE placenta exhibits higher levels of BiP, PERK, and IRE1α in trophoblast layer vs. controls (*n* = 5, *p < 0.05). Data are presented as mean ± SD and analyzed by a Student’s *t* test.

**Fig. 7:**
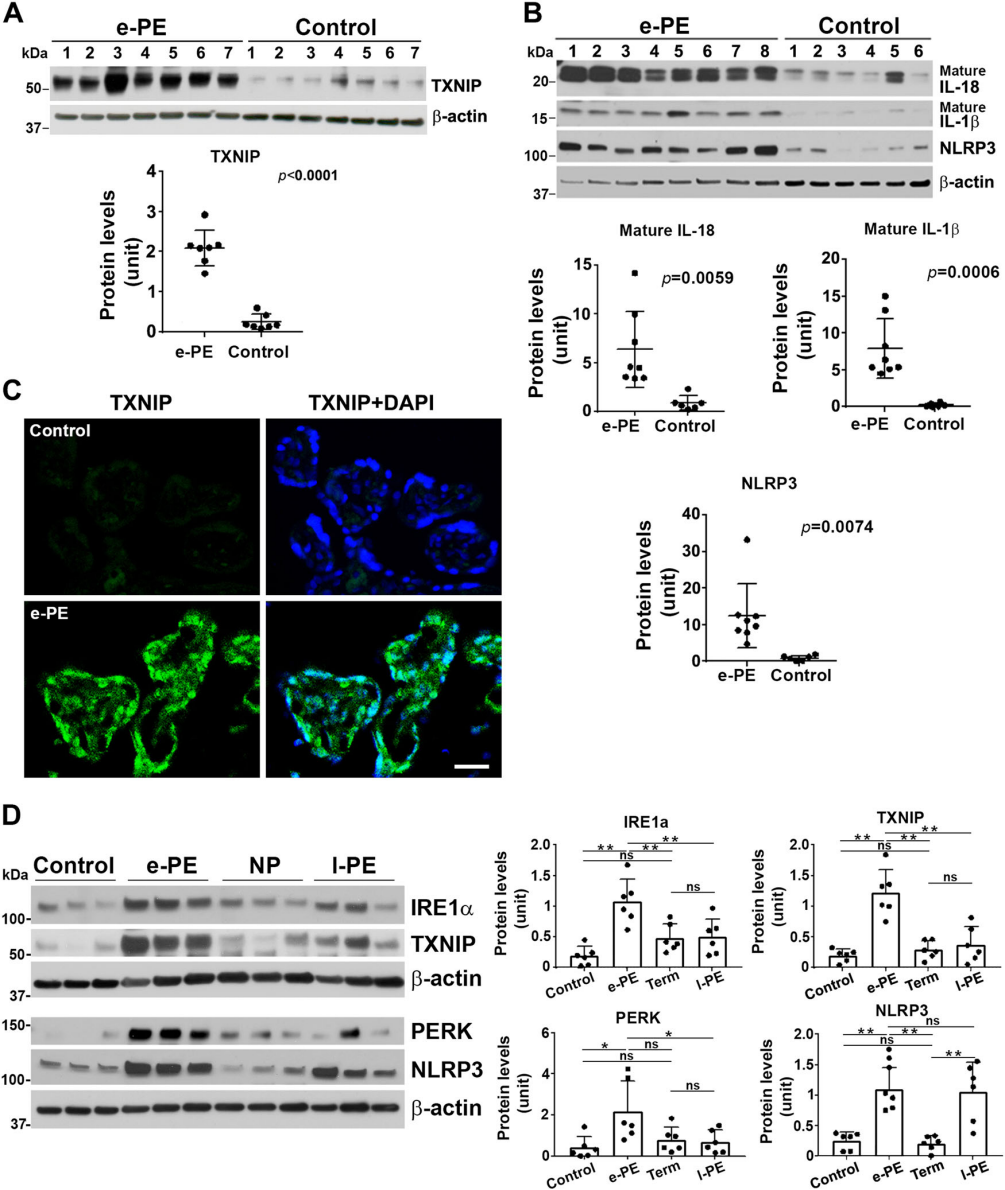
TXNIP, NLRP3, mature IL-18, and IL-1β are elevated in e-PE placenta. **a** Placental protein extract was subjected to western blotting and probed for TXNIP. The expression levels of TXNIP were higher in the placenta from e-PE vs. control (*n* = 7, *p* < 0.05). **b** Immunoblotting shows the expression of mature IL-18, mature IL-1β, and NLRP3 in the placenta from e-PE (*n* = 8) vs. control (*n* = 6). Quantitative analyses show significant increase in indicated molecules in e-PE placenta vs. controls (*p* < 0.05). **c** Representative immunofluorescent images show robust TXNIP expression in trophoblast layer of placenta from e-PE vs. control. Nuclei were stained with DAPI. Images represent five independent placental samples of each group (*n* = 5). Data are presented as mean ± SD and analyzed by a Student’s *t* test. **d** Comparison of IRE1α, PERK, and TXNIP abundance among the placentas from e-PE, l-PE, preterm control, and normal term pregnancy in the same gel. Identical blots in Fig. 1a, b were probed for indicated target molecules. Densitometry of bands was measured and statistically analyzed. Data are presented as mean ± SD and analyzed by a one-way ANOVA. *N* = 6, *p < 0.05; **p < 0.01.

Western blotting analyses revealed higher expression levels of UPR components including BiP, PERK, and IRE1α in the e-PE vs control placental tissue (Fig. 6a, *p* < 0.05). Moreover, similar results were also observed in the e-PE placentas using immunofluorescent staining compared with the control placentas (Fig. 6b). Higher expression levels of these proteins were seen mainly localized to the trophoblast layer of villi (Fig. 6b, *p* < 0.05). Some of these proteins also showed stromal staining, particularly in the case of IRE1α. These results suggest that placental tissue from e-PE display increased UPR. In contrast, the placenta from l-PE displayed at best a mild increase in protein abundance of BiP (1.66 ± 0.48) as compared with NP (1.35 ± 0.48) (Supplementary Fig. 4) as analyzed by western blotting. Statistical analyses, however, revealed no significant difference in BiP expression between l-PE and NP (Supplementary Fig. 4, *p* = 0.247). To evaluate whether downstream pathways are hyperactivated in the e-PE placentas, we next examined protein content of TXNIP and its downstream signaling molecules, including NLRP3, IL-1β, and IL-18. Immunoblotting results demonstrated significant increase in the expression levels of all the proteins listed above (Fig. 7a, b, *p* < 0.05). Significantly higher levels of TXNIP were observed mainly in the trophoblast layer of e-PE placental villi using immunostaining (Fig. 7c).

To exclude the possibility that these target molecules were not increased in the placenta from e-PE, but downregulated in the placenta from preterm control relative to NP, we compared the expression of these proteins in the placentas between preterm control, NP, e-PE, and l-PE on the same gel (Fig. 7d). Again, higher levels of IRE1α, PERK, TXNIP, and NLRP3 were observed in e-PE placentas vs. controls. In contrast, the levels of IRE1α, TXNIP, and PERK expression, albeit a bit higher in NP, and the abundance of NLRP3 expression, although a slightly higher in controls, are not statistically significant between controls and NP. In addition, there are no significant differences in the abundance of IRE1α, TXNIP, and PERK between l-PE and NP (Fig. 7d). However, NLRP3 was highly expressed in l-PE as compared with NP, which supports the data showing higher levels of caspase-1 in l-PE vs. NP in Fig. 1B (Fig. 7d).

Taken together, these data suggest that excessive ER stress and UPR activity and pyroptosis-promoted inflammation cascades occurring predominantly in the e-PE placentas, which recapitulate the findings observed in our cellular model of ER stress using hypoxic stimulation.

## Discussion

**Fig. 8:**
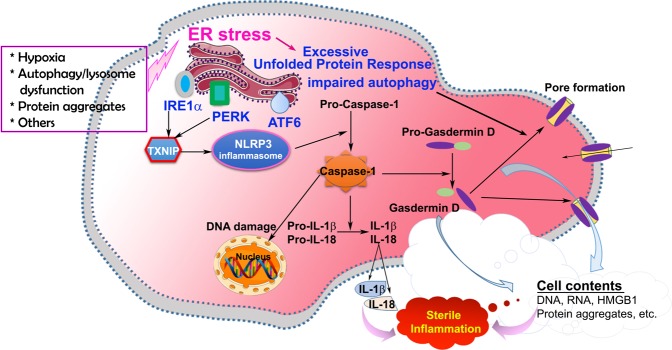
Schematic diagram showing the signaling pathways implicated in regulating pyroptosis in the pathogenesis of PE. Chronic pathological stimuli such as hypoxia induce excessive ER stress, which, in turn, hyperactivates UPR and impairs autophagy. Hyperactivated UPR enhances TXNIP and subsequently triggers NLRP3 inflammasome activation. Activated NLRP3 inflammasome increases the production of active caspase-1, which, in turn, cleaves pro-IL-18/pro-IL-1β and pro-GSDMD into matured IL-18/IL-1β and N-terminal fragments of GSDMD, respectively. N-terminal fragments of GSDMD are translocated to the plasma membrane and form pores, which leads to pyroptosis and subsequent release of cell contents including DAMP, contributing to sterile inflammation. On the other hand, impaired autophagy may trigger pyroptosis, leading to sterile inflammation.

Although inflammation is a well-recognized contributing factor to the multifactorial etiology of PE, the type of inflammation associated with early or late onset PE and the molecular events leading to PE are not yet fully understood. Herein, we provide evidence for the first time that pyroptosis-associated inflammation, as evidenced by increased presence of caspase-1, GSDMD, IL-1β, and IL-18, is readily detected in placental tissue from e-PE deliveries as well as in human trophoblasts exposed to hypoxia, ER stressors, or sera from PE patients. Mechanistically, excessive ER stress and UPR lead to promotion of the NLRP3-pyroptotic inflammatory pathway through TXNIP contributing to sterile inflammation and early onset PE pathology. A schematic model presented in Fig. 8 summarizes our findings culminating into pathways that program inflammatory pyroptosis.

### NLRP3 inflammasome and pyroptosis in PE

The inflammasome is a multiprotein complex which consists of a group of proteins including NOD-like receptor pyrin-containing receptors, ASC, caspase-1, and AIM2-like receptors^24,53,54^. Inflammasome can initiate caspase-1-dependent maturation of the proinflammatory cytokines IL-1β and IL-18 and subsequently elicit pyroptosis^55^. NLRP3 inflammasome has been found to inhibit trophoblast cell differentiation and placental development^24^. Deficiency in NLRP3 activity prevents extracellular vesicles-induced PE-like features in mice^24^. Stimuli such as monosodium urate and LPS can activate NLRP3 inflammasome and increase the production of active caspase-1 and IL-1β in trophoblast cells^56–58^ and monocytes isolated from women with PE^59^. In this study, we found that chronic hypoxic stimulation remarkably upregulates NLRP3, caspase-1, IL-1β, and IL-18 in primary trophoblasts. Notably, protein levels of these molecules were increased in placental tissues from e-PE deliveries, and enhanced NLRP3 and caspase-1 were also observed in l-PE. Our results are consistent with recent studies showing higher immunoreactivity of NLRP3^24,53,54^, caspase-1^53^, and IL-1β^54^ in the trophoblast layer of the placenta from sever PE, although placental tissues were not classified based on e-PE, l-PE, and their corresponding controls in these studies^24,53,54^. It has been suggested that activation of the NLRP3-caspase-1 signaling is a key feature of many diseases^23–25,27,53,54^. Whether alteration of the NLRP3-caspase-1 pathway culminates in pyroptosis in the PE placenta remains unclear.

Pyroptosis was first described as a programmed cell death mode that is initiated by inflammasome-mediated caspase-1 activation in response to infection with bacterial and viral pathogens in innate immune cells, such as monocytes and macrophages^22^. However, growing evidence has demonstrated that pyroptosis also elicits sterile inflammation in nonimmune cells in response to endogenous danger signals^23,25,27,30^. Recently, identification of GSDMD as an essential terminal executor of pyroptotic cell death has significantly enhanced our understanding of the mechanisms for pyroptosis^39–44^. Proteomics studies revealed that GSDMD is cleaved by caspase-1 into N-terminal fragment (p30) and C-terminal fragment (p20)^60^. The N-terminal fragment of GSDMD (NT-GSDMD) translocates to and oligomerize at the plasma membrane and forms pores, which allows the release of cell contents and cytokines to the extracellular matrix as well as influx of fluid into the cytoplasm. Here, we show that NT-GSDMD is significantly upregulated in the placental tissues from women with e-PE not l-PE. Morphological analyses of protein distribution reveal a preferential localization of GSDMD to apical surface of syncytiotrophoblast layer of villi from e-PE placenta. These results were fully recapitulated in autophagy-deficient trophoblasts treated with PES as well as in primary trophoblasts exposed to hypoxia or ER stress inducers. Since autophagy deficiency, hypoxia and ER stress are associated with the pathogenesis of PE^4,8,13–15,33,34,45,46^, our results suggest that these pathological paradigms may contribute to the mechanisms underlying pyroptosis in PE placenta.

A hallmark feature of PE pathophysiology is the impairment of trophoblast invasion of uterine spiral arteries, leading to reduced placental perfusion, ensued hypoxia/ischemia, and nutrition insufficiency^15,16,61–63^. These alterations have been associated with trophoblast cell death^63–66^. Indeed, prior evidence has shown that trophoblast cells in the placenta from preeclamptic pregnancies undergo excessive cell death, including apoptosis^64,65^, necrosis^16^, and necroptosis^21^. Necrosis, necroptosis, and pyroptosis can induce the release of DAMPs, such as microparticles, micro-/nano-vesicles, DNA, RNA, uric acid, and protein aggregates, causing sterile inflammation and oxidative stress in a number of diseases^8,19,20,24^. In this study, however, we found no evidence of intrinsically activated (phosphorylated) MLKL, executor of necroptosis, in placental tissue from e-PE or l-PE deliveries. It is possible that ER stress or hypoxia do not induce activation of MLKL. Nonetheless, our data suggest that pyroptosis in trophoblasts of the e-PE placenta is a unique mechanism for production of DAMPs in the placenta and subsequent release into the maternal circulation.

### TXNIP as a regulatory liaison of the NLRP3 inflammasome-pyroptotic pathway in e-PE

Pathological stimuli such as hypoxia can disrupt ER homeostasis, leading to ER stress and activation of the UPR. The UPR is the initial cellular machinery responsible for clearing misfolded and aggregated proteins to maintain ER homeostasis. However, excessive UPR activation can lead to inflammation and cell death in many disorders, such as autoimmune, infectious, neurodegenerative, and metabolic diseases^25,48,67^. Thus, UPR works as a double-edged sword; either protecting cells under mild ER stress conditions or destroying cells under irremediable ER stress conditions^8^. Our results reveal elevated levels of BiP, PERK, IRE1α, TXNIP, and GSDMD in human primary trophoblasts under persistent hypoxic stimulation as well as in placentas from e-PE, but not l-PE by comparing these molecules among e-PE, l-PE, and their corresponding controls in the same gel (Fig. 7d). This supports the previous observations that increased ER stress and UPR activity were associated with the pathophysiology of e-PE, but not l-PE^8,33,34^, although increased NLRP3 and caspase-1 were also observed in l-PE in our study.

Previous evidence suggests that the UPR and inflammation are intertwined through multiple signaling molecules, including reactive oxygen species, the transcription factor nuclear factor-kB, and the mitogen-activated protein kinase^68^. Notably, in this study, blockade of UPR activity by inhibiting IRE1α and PERK activities reduced protein abundance of TXNIP and caspase-1. Furthermore, inhibition of TXNIP reduced the expression of NLRP3 and caspase-1, and NLRP3 inhibitor decreased caspase-1 abundance in primary human trophoblasts exposed to chronic hypoxia. Our findings suggest that persistent highly elevated levels of PERK and IRE1α induce TXNIP upregulation; TXNIP in turn increases NLRP3 inflammasome, which promotes production of caspapse-1. Although TXNIP may be implicated in NLRP3 priming, but not direct NLRP3 inflammasome activation and other pathways may also contribute to activate NLRP3 inflammasome^69^, our current results point to TXNIP as a regulatory link between hyperactivated UPR to inflammasome and cell death in the e-PE placenta and in trophoblasts exposed to hypoxia-mediated ER stress. The present observations are in agreement with prior studies on β cells^51,52,67^. However, a prior study by Mogami et al. reported that 24 -h treatment of hypoxia suppressed TXNIP expression both at mRNA and protein levels in human placental explants^70^. This discrepancy with our results may be due to difference in time length of hypoxic treatment. In our study, primary human trophoblasts were exposed to hypoxia for 3 days to mimic chronic pathological stimulation of hypoxia. On the other hand, Mogami et al. treated placental explants with hypoxia for 24 h to imitate physiological low oxygen tension in the first trimester^70^. Collectively, TXNIP may serve as a potential target for therapeutic strategy to block switching of excessive UPR activation to deadly inflammatory pathways.

In conclusion, we provide the first compelling evidence that elevated pyroptosis occurs in the trophoblast cells of the placentas from pregnancies complicated with e-PE, and that interconnection of overactivated UPR with NLRP3 inflammasome through TXNIP may contribute to the mechanisms underlying pyroptosis in the placenta from PE women. Pyroptosis serves as a novel cell death mode in the PE placenta, contributing to the release of alarmins and placental debris into maternal circulation which trigger sterile inflammation in the pathogenesis of PE. Therefore, excessive ER stress and UPR activities and pyroptosis can potentiate the inflammatory cascade thought to be responsible for the systemic pathogenesis of PE.

## Supplementary information

Supplementary Figure Legends

Figure S1

Figure S2

Figure S3

Figure S4

Supplementary Table 1

## References

[1] Sibai B, Dekker G, Kupferminc M (2005). Pre-eclampsia. Lancet.

[2] Roberts JM, Redman CW (1993). Pre-eclampsia: more than pregnancy-induced hypertension. Lancet.

[3] Redman CW, Sargent IL (2005). Latest advances in understanding preeclampsia. Science.

[4] Phipps EA, Thadhani R, Benzing T, Karumanchi SA (2019). Pre-eclampsia: pathogenesis, novel diagnostics and therapies. Nat. Rev. Nephrol..

[5] Saade GR (2009). Pregnancy as a window to future health. Obstet. Gynecol..

[6] Smith GC, Pell JP, Walsh D (2001). Pregnancy complications and maternal risk of ischaemic heart disease: a retrospective cohort study of 129,290 births. Lancet.

[7] Cheng SB, Sharma S (2016). Preeclampsia and health risks later in life: an immunological link. Semin. Immunopathol..

[8] Cheng SB, Nakashima A, Sharma S (2016). Understanding pre-eclampsia using Alzheimer’s etiology: an intriguing viewpoint. Am. J. Reprod. Immunol..

[9] Vikse BE, Irgens LM, Leivestad T, Skjaerven R, Iversen BM (2008). Preeclampsia and the risk of end-stage renal disease. N. Engl. J. Med..

[10] Staff AC (2013). Redefining preeclampsia using placenta-derived biomarkers. Hypertension.

[11] Bellamy L, Casas JP, Hingorani AD, Williams DJ (2007). Preeclampsia and risk of cardiovascular disease and cancer in later life: a systemic review and meta-analysis. BMJ.

[12] Steegers EA, von Dadelszen P, Duvekot JJ, Pijnenborg R (2010). Pre-eclampsia. Lancet.

[13] Lai Z, Kalkunte S, Sharma S (2011). A critical role of interleukin-10 in modulating hypoxia-induced preeclampsia-like disease in mice. Hypertension.

[14] Sharma S (2018). Autophagy-based diagnosis of pregnancy hypertension and pre-eclampsia. Am. J. Pathol..

[15] Soleymanlou N (2005). Molecular evidence of placental hypoxia in preeclampsia. J. Clin. Endocrinol. Metab..

[16] Huppertz B (2003). Hypoxia favours necrotic versus apoptotic shedding of placental syncytiotrophoblast into the maternal circulation. Placenta.

[17] Venkatesha S (2006). Soluble endoglin contributes to the pathogenesis of preeclampsia. Nat. Med..

[18] Cheng SB, Davis S, Sharma S (2018). Maternal-fetal cross talk through cell-free fetal DNA, telomere shortening, microchimerism, and inflammation. Am. J. Reprod. Immunol..

[19] Kalkunte SS (2013). Transthyretin is dysregulated in preeclampsia, and its native form prevents the onset of disease in a preclinical mouse model. Am. J. Pathol..

[20] Tong M (2017). Aggregated transthyretin is specifically packaged into placental nano-vesicles in preeclampsia. Sci. Rep..

[21] Bailey LJ, Alahari S, Tagliaferro A, Post M, Caniggia I (2017). Augmented trophoblast cell death in preeclampsia can proceed via ceramide-mediated necroptosis. Cell Death Dis..

[22] Brennan MA, Cookson BT (2000). Salmonella induces macrophage death by caspase-1-dependent necrosis. Mol. Microbiol..

[23] Chen A (2018). Liraglutide attenuates NLRP3 inflammasome-dependent pyroptosis via regulating SIRT1/NOX4/ROS pathway in H9c2 cells. Biochem. Biophys. Res. Commun..

[24] Kohli S (2016). Maternal extracellular vesicles and platelets promote preeclampsia via inflammasome activation in trophoblasts. Blood.

[25] Lebeaupin C (2015). ER stress induces NLRP3 inflammasome activation and hepatocyte death. Cell Death Dis..

[26] Sun N, Zhang H (2018). Pyroptosis in pterygium pathogenesis. Biosci. Rep..

[27] Vakrakou AG (2018). Systemic activation of NLRP3 inflammasome in patients with severe primary Sjögren’s syndrome fueled by inflammagenic DNA accumulations. J. Autoimmun..

[28] Bergsbaken T, Fink SL, Cookson BT (2009). Pyroptosis: host cell death and inflammation. Nat. Rev. Microbiol..

[29] Fink SL, Cookson BT (2005). Apoptosis, pyroptosis, and necrosis: mechanistic description of dead and dying eukaryotic cells. Infect. Immun..

[30] Heo MJ (2018). Alcohol dysregulates miR-148a in hepatocytes through FoxO1, facilitating pyroptosis via TXNIP overexpression. Gut.

[31] Vande WalleL, Lamkanfi M (2016). Pyroptosis. Curr. Biol..

[32] Roberts JM (2013). Hypertension in pregnancy, report of the American college of obstetricians and gynecologists’ task force on hypertension in pregnancy. Obstet. Gynecol..

[33] Yung HW (2014). Differential activation of placental unfolded protein response pathways implies heterogeneity in causation of early- and late-onset pre-eclampsia. J. Pathol..

[34] Burton GJ, Yung HW (2011). Endoplasmic reticulum stress in the pathogenesis of early-onset pre-eclampsia. Pregnancy Hypertens..

[35] Fujita N (2008). An Atg4B mutant hampers the lipidation of LC3 paralogues and causes defects in autophagosome closure. Mol. Biol. Cell..

[36] Kliman HJ, Nestler JE, Sermasi E, Sanger JM, Strauss JF (1986). Purification, characterization, and in vitro differentiation of cytotrophoblasts from human term placentae. Endocrinology.

[37] Schaiff WT (2005). Peroxisome proliferator-activated receptor-gamma and retinoid X receptor signaling regulate fatty acid uptake by primary human placental trophoblasts. J. Clin. Endocrinol. Metab..

[38] Nelson DM, Johnson RD, Smith SD, Anteby EY, Sadovsky Y (1999). Hypoxia limits differentiation and up-regulates expression and activity of prostaglandin H synthase 2 in cultured trophoblast from term human placenta. Am. J. Obstet. Gynecol..

[39] He WT (2015). Gasdermin D is an executor of pyroptosis and required for interleukin-1β secretion. Cell Res..

[40] Liu X (2016). Inflammasome-activated gasdermin D causes pyroptosis by forming membrane pores. Nature.

[41] Sborgi L (2016). GSDMD membrane pore formation constitutes the mechanism of pyroptotic cell death. EMBO J..

[42] Shi J, Gao W, Shao F (2017). Pyroptosis: gasdermin-mediated programmed necrotic cell death. Trends Biochem Sci..

[43] Rühl S, Broz P (2016). The gasdermin-D pore: executor of pyroptotic cell death. Oncotarget.

[44] Chen X (2016). Pyroptosis is driven by non-selective gasdermin-D pore and its morphology is different from MLKL channel-mediated necroptosis. Cell Res..

[45] Nakashima A (2013). Impaired autophagy by soluble endoglin, under physiological hypoxia in early pregnant period, is involved in poor placentation in preeclampsia. Autophagy.

[46] Nakashima A (2017). Autophagy regulation in preeclampsia: pros and cons. J. Reprod. Immunol..

[47] Kalkunte S (2010). Sera from preeclampsia patients elicit symptoms of human disease in mice and provide a basis for an in vitro predictive assay. Am. J. Pathol..

[48] Cao SS, Luo KL, Shi L (2016). Endoplasmic reticulum stress interacts with inflammation in human diseases. J. Cell Physiol..

[49] Garg AD (2012). ER stress-induced inflammation: does it aid or impede disease progression?. Trends Mol. Med..

[50] Anthony TG, Wek RC (2012). TXNIP switches tracks toward a terminal UPR. Cell Metab..

[51] Lerner AG (2012). IRE1α induces thioredoxin-interacting protein to activate the NLRP3 inflammasome and promote programmed cell death under irremediable ER stress. Cell Metab..

[52] Oslowski CM (2012). Thioredoxin-interacting protein mediates ER stress-induced β cell death through initiation of the inflammasome. Cell Metab..

[53] Weel IC (2017). Increased expression of NLRP3 inflammasome in placentas from pregnant women with severe preeclampsia. J. Reprod. Immunol..

[54] Stødle GS (2018). Placental inflammation in pre-eclampsia by Nod-like receptor protein (NLRP)3 inflammasome activation in trophoblasts. Clin. Exp. Immunol..

[55] Rathinam VA, Fitzgerald KA (2016). Inflammasome complexes: emerging mechanisms and effector functions. Cell.

[56] Mulla MJ (2011). Uric acid induces trophoblast IL-1beta production via the inflammasome: implications for the pathogenesis of preeclampsia. Am. J. Reprod. Immunol..

[57] Pontillo A (2013). Bacterial LPS differently modulates inflammasome gene expression and IL-1beta secretion in trophoblast cells, decidual stromal cells, and decidual endothelial cells. Reprod. Sci..

[58] Tamura K (2017). Glibenclamide inhibits NLRP3 inflammasome-mediated IL-1beta secretion in human trophoblasts. J. Pharm. Sci..

[59] Romão-Veiga M (2018). Induction of systemic inflammation by hyaluronan and hsp70 in women with pre-eclampsia. Cytokine.

[60] Agard NJ, Maltby D, Wells JA (2010). Inflammatory stimuli regulate caspase substrate profiles. Mol. Cell Proteom..

[61] Chappell L, Bewley S (1998). Pre-eclamptic toxaemia: the role of uterine artery Doppler. Br. J. Obstet. Gynaecol..

[62] Kingdom JC, Kaufmann P (1997). Oxygen and placental villous development: origins of fetal hypoxia. Placenta.

[63] Nadeau-Vallée M (2016). Sterile inflammation and pregnancy complications: a review. Reproduction.

[64] Crocker IP, Cooper S, Ong SC, Baker PN (2003). Differences in apoptotic susceptibility of cytotrophoblasts and syncytiotrophoblasts in normal pregnancy to those complicated with preeclampsia and intrauterine growth restriction. Am. J. Pathol..

[65] Longtine MS, Chen B, Odibo AO, Zhong Y, Nelson DM (2012). Villous trophoblast apoptosis is elevated and restricted to cytotrophoblasts in pregnancies complicated by preeclampsia, IUGR, or preeclampsia with IUGR. Placenta.

[66] Cali U, Cavkaytar S, Sirvan L, Danisman N (2013). Placental apoptosis in preeclampsia, intrauterine growth retardation, and HELLP syndrome: an immunohistochemical study with caspase-3 and bcl-2. Clin. Exp. Obstet. Gynecol..

[67] Zhou R, Tardivel A, Thorens B, Choi I, Tschopp J (2010). Thioredoxin-interacting protein links oxidative stress to inflammasome activation. Nat. Immunol..

[68] Zhang K, Kaufman RJ (2008). From endoplasmic-reticulum stress to the inflammatory response. Nature.

[69] Elliott EI, Sutterwala FS (2015). Initiation and perpetuation of NLRP3 inflammasome activation and assembly. Immunol. Rev..

[70] Mogami H (2017). Differential expression of thioredoxin binding protein-2/Txnip in human placenta: Possible involvement of hypoxia in its suppression during early pregnancy. J. Obstet. Gynaecol. Res..

