# Supplementary material for: Pyroptosis is a critical inflammatory pathway in the placenta from early onset preeclampsia and in human trophoblasts exposed to hypoxia and endoplasmic reticulum stressors
Source: Cell Death Dis. 2019 Dec 5;10(12):927. doi: 10.1038/s41419-019-2162-4 (PMC6895177; doi:10.1038/s41419-019-2162-4)
Supplement: Supplementary file 1 — Supplementary Figure Legends [file 41419_2019_2162_MOESM1_ESM.doc]

**Supplementary Figures**

**Supplementary Fig. 1 Expression of GSDMD and caspase-1 in the placentas from e-PE, l-PE, preterm birth (control) and normal term pregnancy (NP). a-c,** Placental protein extracts were subjected to western blotting. The blots were probed for GSDMD, caspase-1 and β-actin. Statistical analyses revealed no significant difference in GSDMD between L-PE and NP placentas (n=7). Blots of caspase-1 and GSDMD were probed from identical membrane used in Figure 6a and Figure 8a, respectively. Data are presented as mean ± S.D. and analyzed by a Student *t*-test.

**Supplementary Fig. 2 Detection of total MLKL and phosphorylated MLKL (pMLKL) expression in the placentas from e-PE and preterm birth control. a** Western blots of placental protein extracts were probed for total MLKL, pMLKL and β-actin. Quantitative analyses showed no significant difference in total MLKL abundance between e-PE and control placentas. (n=7, *p*=0.797). **b** pMLKL was not detected in any of the e-PE or control placentas but was detected in the positive control (PC, provided by Cell Signaling) from HT-29 cells treated with combination of Z-VAD (20 μM, added 30 min prior to other compounds), human TNF-α (20 ng/ml, 7 hr) and SM-164 (100 nM, 7 hr), and necrostatin (50 μM, 7 hr). Data are presented as mean ± S.D. and analyzed by a Student *t*-test.

**Supplementary Fig. 3 GSDMD is upregulated in human primary trophoblast cells treated with ER stress inducers. a** Primary trophoblast cells were treated with vehicle, BFA or chloroquine overnight, fixed and immunostained for GSDMD. Arrows indicate the localization of GSDMD to the cell surface. Images are representatives of three independent experiments. **b** Quantitative analysis revealed that reagent-treated cells had higher levels of GSDMD than vehicle-treated cells (vehicle: n=21; BFA: n=26; chloroquine: n=31, *p*<0.05). Data are presented as mean ± S.D. and analyzed by a Student *t*-test.

**Supplementary Fig. 4 Comparison of BiP abundance in the placentas between l-PE vs. NP.** Placental protein extracts were subjected to immunoblotting analyses. Identical membrane used in supplementary Figure 1b was re-probed for BiP. Although l-PE placentas exhibited a slight increase in the content of this protein, statistical analyses revealed no significant difference between l-PE and NP placentas (n=7, *p*=0.247). Data are presented as mean ± S.D. and analyzed by a Student *t*-test.
