# Supplementary figures and images for: Pyroptosis is a critical inflammatory pathway in the placenta from early onset preeclampsia and in human trophoblasts exposed to hypoxia and endoplasmic reticulum stressors
Source: Cell Death Dis. 2019 Dec 5;10(12):927. doi: 10.1038/s41419-019-2162-4 (PMC6895177; doi:10.1038/s41419-019-2162-4)

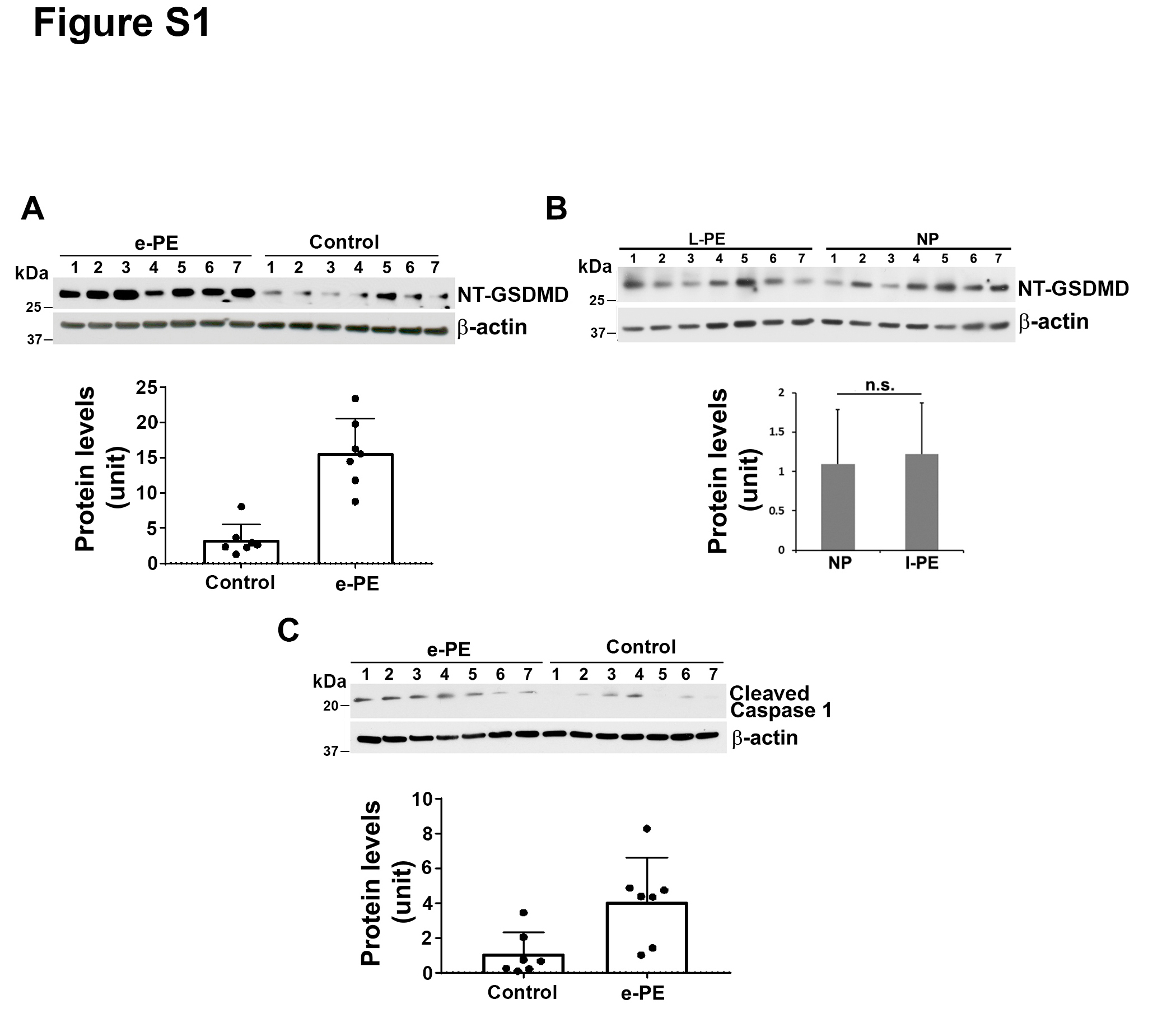

Supplement: Supplementary file 2 — Figure S1 [file 41419_2019_2162_MOESM2_ESM.tif]

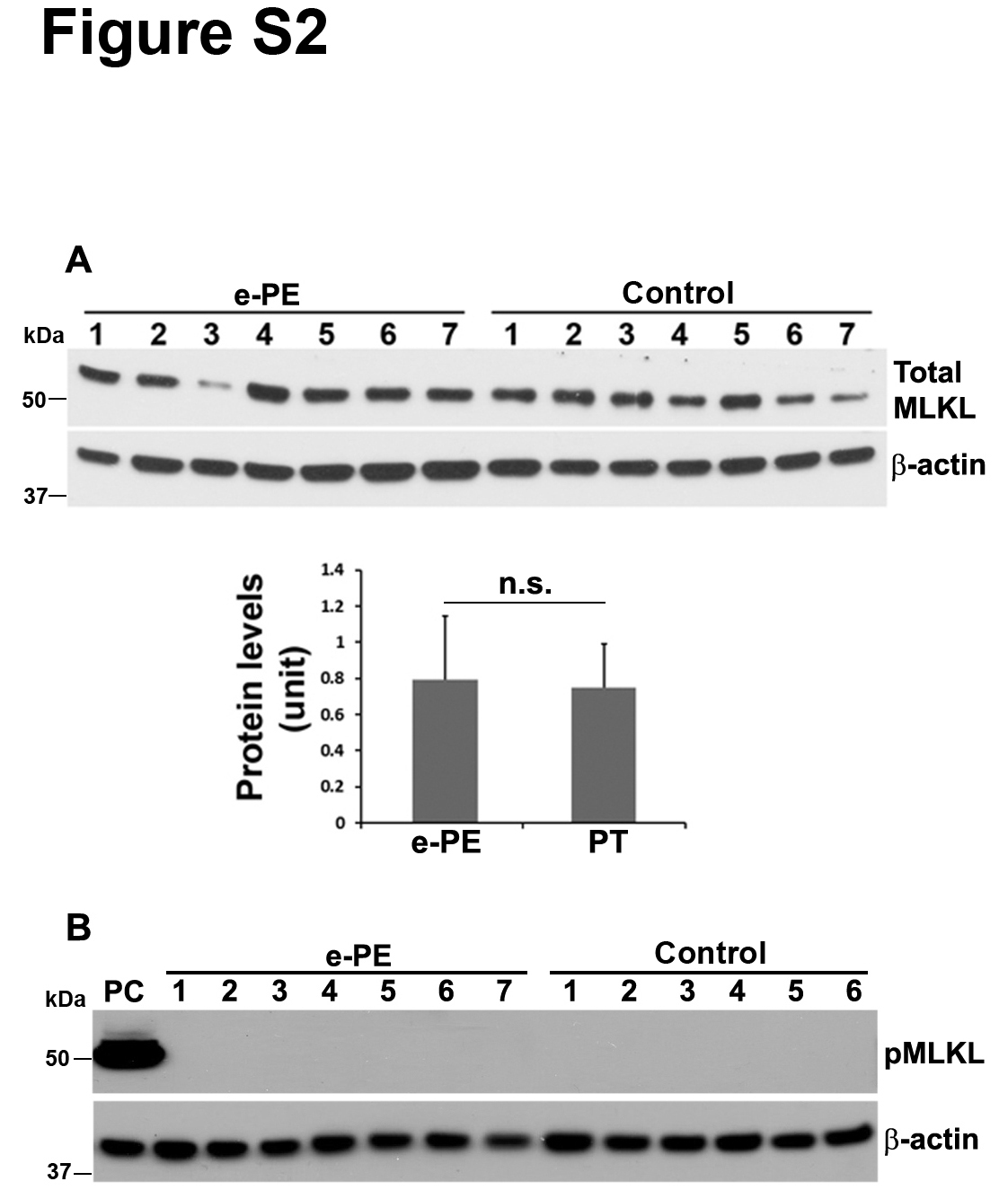

Supplement: Supplementary file 3 — Figure S2 [file 41419_2019_2162_MOESM3_ESM.tif]

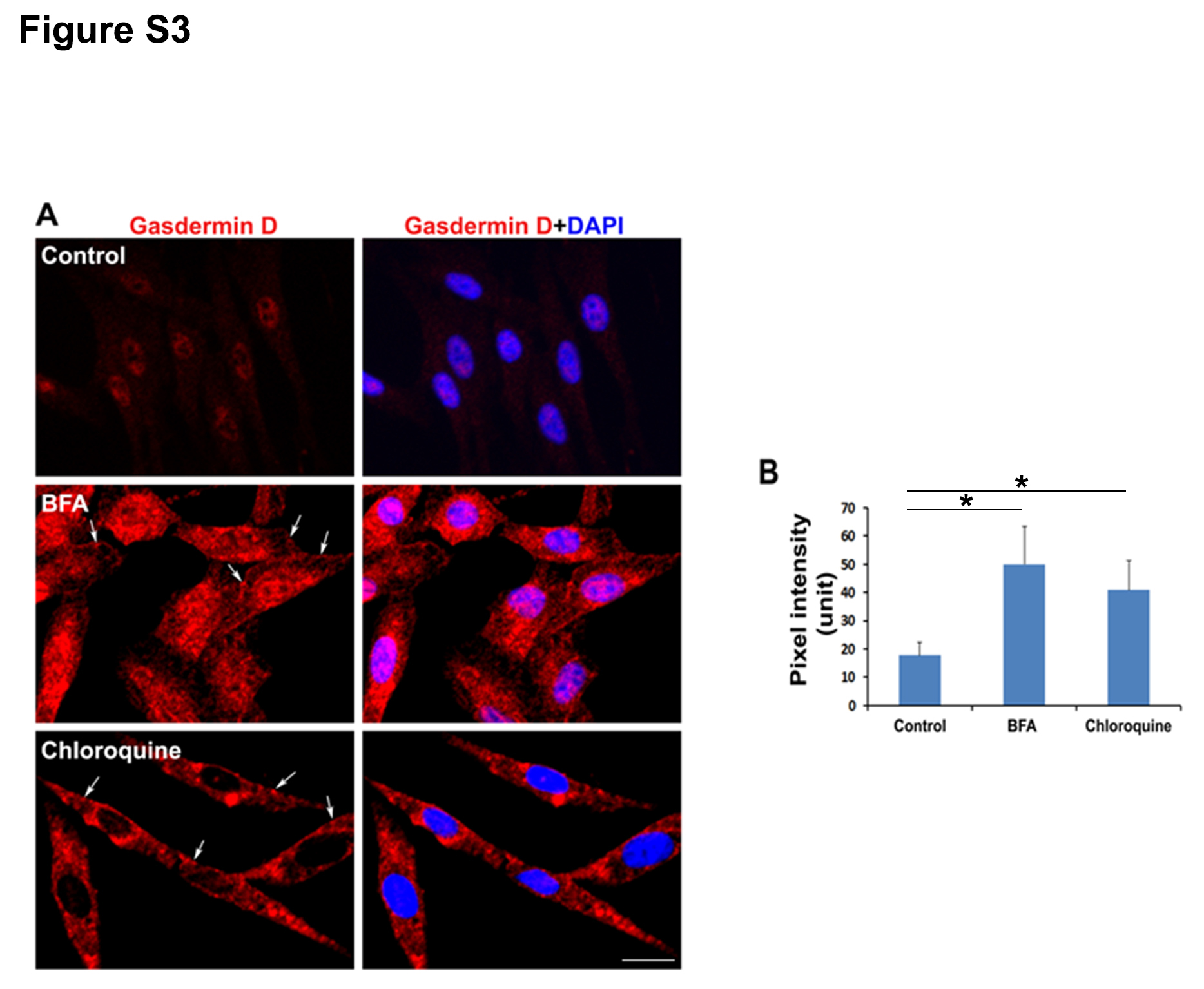

Supplement: Supplementary file 4 — Figure S3 [file 41419_2019_2162_MOESM4_ESM.tif]

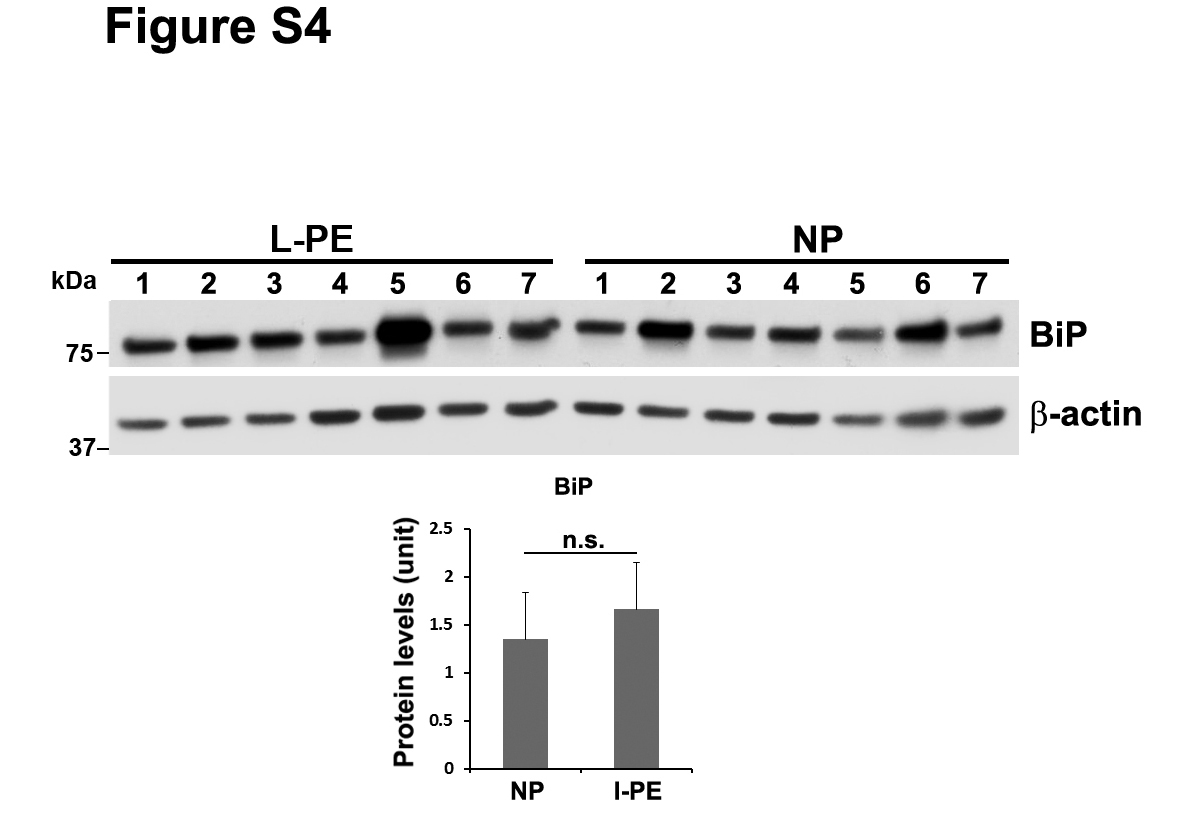

Supplement: Supplementary file 5 — Figure S4 [file 41419_2019_2162_MOESM5_ESM.tif]
