# Supplementary material for: Pyroptosis is a critical inflammatory pathway in the placenta from early onset preeclampsia and in human trophoblasts exposed to hypoxia and endoplasmic reticulum stressors
Source: Cell Death Dis. 2019 Dec 5;10(12):927. doi: 10.1038/s41419-019-2162-4 (PMC6895177; doi:10.1038/s41419-019-2162-4)
Supplement: Supplementary file 6 — Supplementary Table 1 [file 41419_2019_2162_MOESM6_ESM.doc]

**Supplementary Table**

**Table S1:** **Demographic and clinical characteristics of patients**

| Variable | Late onset preeclampsia with severe features  (n=10) | Normal term pregnancy  (n=10) | Early onset preeclampsia  (n=8) | Pre-term birth control  (n=8) | *p*-value |
| --- | --- | --- | --- | --- | --- |
| Age (years) | 30.3 (9.4) | 29.3 (7.2) | 29.8 (7.1) | 24.8 (5.8) | † 0.41^1^  †† 0.14^1^ |
| Race  White  Black  Hispanic  Other | 5 (50%)  3 (30%)  2 (20%)  0 | 7 (70%)  2 (20%)  1 (10%)  0 | 4 (50%)  1 (12.5%)  3 (37.5%)  0 | 3 (37.5%)  2 (25%)  3 (37.5%)  0 | †0.51^2^  †† 0.84^2^ |
| BMI (kg/m^2^) | 30.7 (5.4) | 27.9 (4.2) | 32.0 (6.2) | 31.5 (7.2) | † 0.10^1^  †† 0.87^1^ |
| Gestational age at delivery (weeks) | 38.1 (0.77) | 38.4 (1.4) | 31.7 (1.5) | 33.1 (1.3) | † 0.73^3^  †† 0.06^3^ |
| Maternal temperature (°C) | 37.0 (0.25) | 36.9 (0.2) | 36.9 (0.13) | 36.8 (0.18) | † 0.23^1^  †† 0.36^1^ |
| Maximum systolic blood pressure (mmHg) | 171 (12.8) | 118 (9.5) | 179 (13.2) | 122 (13.8) | † < 0.001^3^  ††<0.001^3^ |
| Maximum diastolic blood pressure (mmHg) | 101 (2.4) | 74.7 (5.7) | 113 (5.9) | 74.6 (5.8) | † < 0.001^3^  ††<0.001^3^ |
| Mode of delivery  Vaginal  Cesarean Section | 6 (60%)  4 (40%) | 7 (70%)  3 (30%) | 3 (37.5%)  5 (62.5%) | 5 (62.5%)  3 (37.5%) | † 0.5^2^  †† 0.62^2^ |
| Maternal hemoglobin (g/dl) | 10.4 (1.5) | 11.3 (0.8) | 10.4 (0.9) | 10.5 (1.6) | † 0.32^1^  †† 0.81^1^ |
| Maternal platelets (x10^3^/µl) | 157 (27.7) | 226 (62.1) | 101 (68.3) | 195 (71.2) | † 0.05 ^1^  †† 0.02^1^ |
| AST (U/L) | 42.3 (14.9) | 11 – 30* | 247 (288) | 11 – 30* | N/A |
| Serum creatinine (mg/dl) | 0.83 (0.16) | 0.5 – 1.1* | 0.97 (0.25) | 0.5 – 1.1* | N/A |
| Urine protein : creatinine | 3.26 (3.11) | < 0.3* | 5.0 (6.6) | < 0.3* | N/A |
| Birth weight (grams) | 2882 (445) | 3227 (713) | 1334 (129) | 2133 (382) | † 0.11^1^  ††<0.001^1^ |

Data presented as Mean (standard deviation) for continuous variables

Data presented as n (%) for categorical variables

*AST, serum creatinine, and urine protein:creatinine not measured in Control subjects and presented as normal ranges.

^1^t-test

^2^ Fisher’s exact test

^3^ Wilcoxon rank-sum

†late onset preeclampsia with severe features versus term controls

†† early onset preeclampsia with severe features versus preterm controls
